# Evaluation of the Effects of Eye Drops for Dry Eyes on Neuronal Pain Receptors in a Primary Culture Model of Trigeminal Ganglion Cells

**DOI:** 10.3390/jcm14228038

**Published:** 2025-11-13

**Authors:** Chihiro Sunouchi, Takahiko Hayashi, Satoru Yamagami, Tohru Sakimoto

**Affiliations:** 1 Department of Ophthalmology, Nihon University School of Medicine, Tokyo 173-8610, Japan; sunouchi.chihiro@nihon-u.ac.jp (C.S.); yamagami.satoru@nihon-u.ac.jp (S.Y.); 2 Sugiura Eye Clinic, Shizuoka 416-0955, Japan; torusaki@gmail.com

**Keywords:** dry eye, ocular surface, primary culture model, trigeminal ganglion cell

## Abstract

**Background:** Dry eye disease is a multifactorial ocular surface disorder characterized by tear film instability, inflammation, and neurosensory abnormalities that can lead to corneal pain and discomfort. In this study, we evaluated the effects of specific eye drops for dry eyes on neuronal pain receptors to gain insight into the mechanisms underlying corneal nerve pain in patients with dry eyes using a primary cell culture model of murine trigeminal ganglion cells. **Methods:** Trigeminal ganglia were obtained from wild-type postnatal day 7–10 mice. Primary cultures were prepared using the cell suspension method. After culturing for one week, the cells were stained with neuron-specific anti-neuronal nuclei, polymodal nociceptor, and transient receptor potential vanilloid 1 (TRPV1) antibodies. The calcium ion probe Fura2-AM^®^ was added to cultured cells after 2 weeks of incubation. The effects of capsaicin alone, in combination with the TRPV1 antagonist AMG9810, and in the presence of components of commercially available eye drops (cyclosporine, diquafosol tetrasodium, or rebamipide) were evaluated by monitoring calcium signals. **Results:** Neural excitation and capsaicin-induced increase in fluorescence intensity ratio were suppressed by AMG9810, cyclosporine, and diquafosol tetrasodium, but not by rebamipide. **Conclusions:** Inhibition of cellular excitation by cyclosporine and diquafosol tetrasodium may underlie their clinical pain suppressive effects. The primary culture model described here may serve as a useful tool for future studies on corneal perception.

## 1. Introduction

Dry eye is a multifactorial disease affecting the ocular surface, characterized by loss of tear film homeostasis, with ocular symptoms in which tear film instability and hyperosmolarity, ocular surface inflammation and damage, and neurosensory abnormalities play etiological roles [1].

However, clinical aspects of the condition—including diagnosis of a corneal epithelial disorder or low lacrimal fluid volume—do not always correlate with subjective symptoms such as pain and foreign body sensation. Additionally, after mechanical, thermal, or chemical stimulation, patients with dry eyes exhibit corneal paresthesia, which is potentially associated with corneal nerve damage [2].

The ophthalmic nerve is the first division of the trigeminal nerve that supplies sensory innervation to the cornea, which has a high density of sensory nerves and is 300–400 times more sensitive than the fingers or tooth pulp. Therefore, even slight corneal damage can cause a foreign body sensation and induce severe pain [3]. The corneal nerve has polymodal nociceptors and mechanoreceptors that perceive mechanical stimulation, as well as cold nociceptors that sense cold temperature [4]. Nociceptive pain is perceived by a sensory nerve following substantial injury involving inflammation in non-neural tissues.

Evaluation of nociceptor sensitivity can identify the mechanisms underlying pain perception by the corneal nerve; however, mouse models to evaluate nociceptor sensitivity are currently lacking [5,6,7,8,9,10,11,12]. Therefore, in this study, we evaluated the sensitivity of polymodal corneal nociceptors using cultured trigeminal ganglion-derived cells (TRGCs) from mice and examined the nociceptor-specific transient receptor potential vanilloid 1 (TRPV1) responses. Furthermore, we investigated whether current clinical medications exert excitatory or inhibitory effects on these cells.

## 2. Materials and Methods

### 2.1. Animals

Pregnant C57BL/6J mice were purchased from Charles River Laboratories Japan, Inc. (Tokyo, Japan). Young mice aged 7–10 days were used for experiments. Animals were treated in accordance with the animal experiment protocol approved by the Animal Experiment Committee of Nihon University School of Medicine (approval number: AP20MED040-1). All methods were performed in accordance with relevant guidelines and regulations. Furthermore, this study was conducted in accordance with the ARRIVE guidelines (https://arriveguidelines.org). Pregnant mice were housed together with their offspring in the same cage until used for experiments. The group allocation was managed by SY.

### 2.2. Primary Culture of Trigeminal Ganglion Neurons

Mice were cervically dislocated and decapitated under hypothermia, without anesthesia, by skilled technicians. After sterilization with 0.5% povidone-iodine, the scalp was removed. The tissue was rinsed three times with Hank’s Balanced Salt Solution (HBSS; FUJIFILM Wako Pure Chemical Corp., Osaka, Japan). The trigeminal ganglia—visible as white, straight-line tissue on the cranial base after opening the skull—were removed from the exposed brain tissue. All procedures were performed on a clean bench. Each trigeminal ganglion was placed in a non-coated 35 mm dish containing 2 mL HBSS and minced using spring-handle scissors. The tissue was transferred into STEMFULL^®^ centrifuge tubes (Sumitomo Bakelite Co., Ltd., Tokyo, Japan), which reduce cell adhesion to tube walls, and centrifuged for 5 min at 800–1000× *g*. Neural cells were dispersed using a neural cell dispersion solution set (DS Pharma Biomedical Co., Ltd., Osaka, Japan Cat. No. MB-X9901D), which includes enzyme, dispersion, and removal solution. The supernatant was removed, enzyme solution was added, and the samples were incubated at 37 °C for 30 min. After gentle pipetting, samples were centrifuged again for 5 min at 800–1000× *g*. The supernatant was removed, dispersion solution was added, and samples were pipetted again. Removal solution was then slowly added from the bottom of the centrifuge tube to form two layers with the dispersion solution. The volume of each solution was adjusted according to the number of mice and the amount of tissue collected.

**Figure 1:**
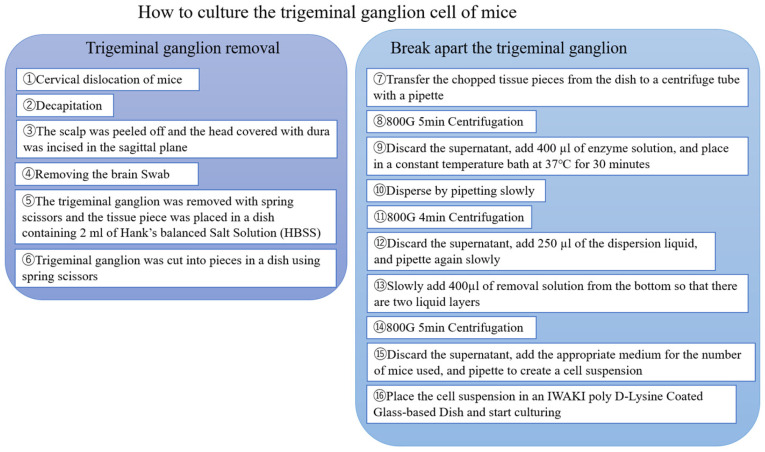
Culturing murine trigeminal ganglion cells. This flowchart shows the process of culturing murine trigeminal ganglion cells in two sections.

Following a final centrifugation (5 min at 800–1000× *g*), the supernatant was removed, and neural cell culture medium (FUJIFILM Wako Pure Chemical Corp., Osaka, Japan; Cat. No. 148-09671) was added to prepare the neural cell suspension, which was then seeded into wells for culture. The suspension was filtered through pluriStrainer Mini 70 μm filters (pluriSelect Life Science, Leipzig, Germany) and distributed into poly-D-lysine–coated plastic cell culture dishes (Iwaki, ACG Techno Glass Co., Ltd., Shizuoka, Japan). The culture medium was replaced every other day. From the second exchange (day 4), 0.1% antibiotic–antimycotic solution (Gibco, Thermo Fisher Scientific, Waltham, MA, USA) was added. To prevent cell aggregation and promote neurite growth, the cell suspension was filtered through a 70-µm strainer. Medium changes were performed gently to avoid cell detachment, as the cells adhered directly to the dish surface. The cells were non-proliferating and therefore could not be sub-cultured. A flowchart of the culture process is shown in Figure 1.

### 2.3. Immunofluorescence Staining

Primary trigeminal ganglion cell cultures (PTGCs) were fixed in 4% paraformaldehyde/phosphate-buffered saline (PBS) for 30 min at room temperature in a 35 mm dish. After washing twice with Dulbecco’s PBS (D-PBS; FUJIFILM Wako Pure Chemical Corp., Osaka, Japan), the cultures were blocked in 10% goat serum for 30 min at room temperature. PTGCs were incubated overnight at 4 °C in the dark with rabbit anti-TRPV1 (1:250; ab3487, polyclonal; Abcam, Cambridge, UK) and rabbit anti-neuronal nuclei (NeuN) (1:500; ab177487, monoclonal; Abcam, Cambridge, UK) antibodies [13,14]. After three washes with D-PBS, the cultures were incubated with Alexa Fluor^TM^ 488 goat anti-rabbit IgG (H + L) (1:200; Thermo Fisher Scientific K.K., Tokyo, Japan) for 90 min at room temperature in the dark. Finally, cultures were counterstained with 4′,6-diamidino-2-phenylindole (DAPI Fluoromount-G, SouthernBiotech, Birmingham, AL, USA), and staining was observed under a fluorescence microscope (BZ-X Serises, Keyence Corp., Osaka, Japan).

### 2.4. Calcium Imaging of Primary Cultures

To evaluate cell excitation, primary cultures were incubated with 5 μM of Fura-2AM^®^ (Invitrogen, Carlsbad, CA, USA), a calcium ion probe [15,16], in 0.04% Pluronic F-127 (COSMO BIO Co., Ltd., Tokyo, Japan) [17,18] at 37 °C under 6% CO_2_ for 60 min. Cultures were then washed twice with HBSS. HBSS, cyclosporine [19,20,21] (10 and 100 µM), rebamipide [22,23,24,25] (10 and 100 µM), diquafosol tetrasodium (1, 10, and 100 µM), or AMG9810 [26,27,28,29,30,31] (1, 10, and 100 µM; a TRPV1 antagonist) were added to each dish. Continuous images were acquired, and fluorescence intensity was measured with a microplate reader (Infinite^®^ 200 PRO; Tecan Group Ltd., Männedorf, Switzerland) before and after stimulation with 10 µM of capsaicin. The excitation wavelengths were 340 nm and 380 nm, and the emission wavelength was 510 nm. Capsaicin acts on TRPV1, allowing calcium ions to enter the cell, which subsequently activates anoctamin 1. Because sensory nerves have high intracellular chloride concentrations, the cells release chloride ions, generating an action potential [32].

### 2.5. Cell Viability

The viability of TRGCs was assessed using the Cell Counting Kit-8 (CCK-8; Dojindo Laboratories, Kumamoto, Japan) according to the manufacturer’s instructions. Cells were seeded into 24-well plates, and 400 μL of culture medium containing 40 μL of CCK-8 solution was added to each well, followed by incubation for 24 h. Absorbance was measured at 450 nm using a microplate reader (Infinite^®^ 200 PRO; Tecan Group Ltd., Männedorf, Switzerland).

### 2.6. Statistical Analysis

Statistical analyses were performed using GraphPad Prism version 9.1.2 (GraphPad Software, San Diego, CA, USA). Dunnett’s multiple comparisons were performed following one-way analysis of variance, with the Brown–Forsythe applied to account for groups with different sample sizes. Multiple culture experiments were conducted to ensure each group had at least *n* = 3 or more replicates. The sample size was minimized according to the principles of the 3Rs.

## 3. Results

### 3.1. Establishment of PTGCs

We evaluated the following factors to improve cell growth: (1) the age of the mice, (2) contamination prevention methods, and (3) the coating applied to culture dishes and wells.

Although previous studies used mice that were several weeks or months old, they failed to obtain robust cultures of neuronal cells. In contrast, cells isolated from postnatal day 7 mice yielded optimal TRGCs. Povidone-iodine during tissue removal was essential for contamination prevention during culture. Among the various coating materials available to culture neural cells, we selected poly-D-lysine-coated dishes or wells based on previous findings [7,8,9].

**Figure 2:**
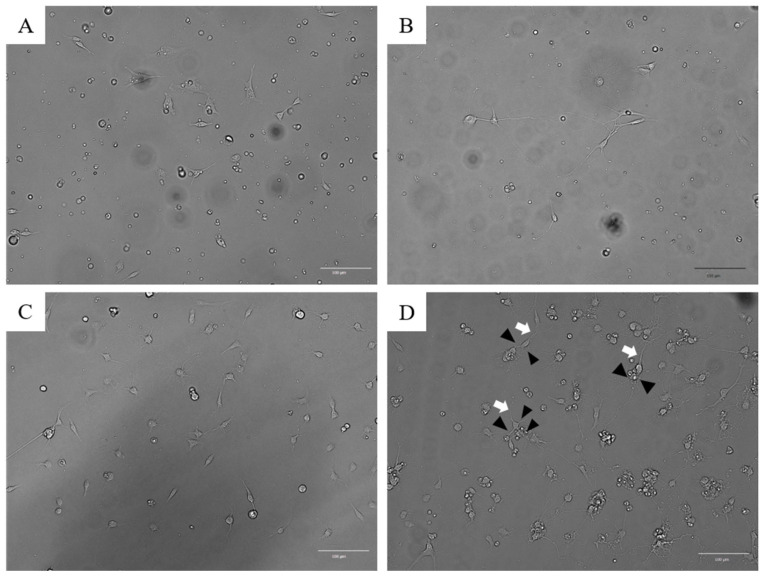
Microscopic images of the developmental process in primary cultures of mouse trigeminal ganglion cells. (**A**) Attached cells on day 1. Attached cells are observed throughout the culture dish, but dendrites and axons are absent; (**B**) Neurites from attached cells on day 2. Some of the cells had neurites, whereas most did not; (**C**) Cell culture on day 7. The dendrites are stretched out like a mesh (white arrow); (**D**) Cell culture on day 11. One long axon and many short dendritic spines were found in neurons (the white arrow and black arrowheads indicate the axon and dendrites, respectively).

Cell growth was monitored using microscopy. TRGCs remained attached to the bottom of the dishes on days 1 and 2 after dispersion (Figure 2A,B). However, cells that remained in suspension during this period rarely adhered later. The adherent cells extended dendrites and axons (Figure 2B), although they did not undergo cell division. After 1–2 weeks of culture, dendrites formed a mesh-like network (Figure 2C). The degree of cell adhesion observed within 2–3 days after plating was predictive of subsequent growth. Approximately 80% of adherent TRGCs developed neurites by day 7. After 1–2 weeks of culture, these cells differentiated into neuron-like cells, each bearing a single long axon and multiple dendrites (Figure 2D).

### 3.2. Immunofluorescence Staining with Anti-NeuN and Anti-TRPV1 Antibodies

**Figure 3:**
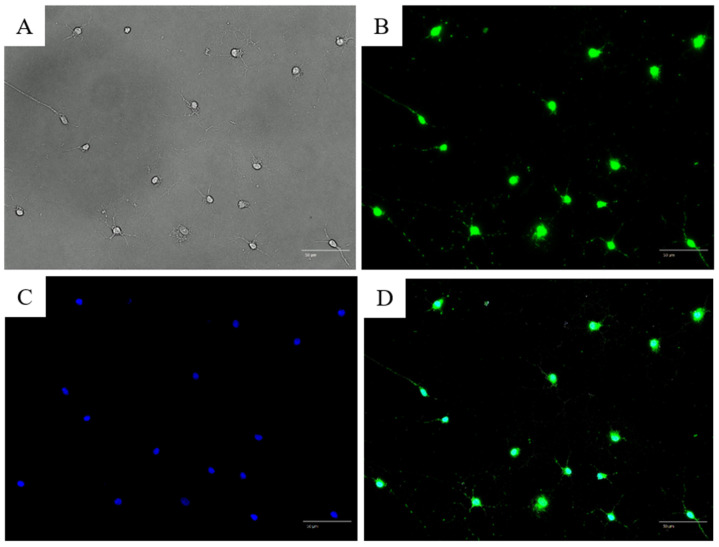
Microscopic images of trigeminal ganglion cells stained with anti-neuronal nuclei antibody after 1 week of culture. (**A**) Bright-field image. One long, thick axon and several dendritic spines are shown; (**B**) Anti-neuronal nuclei (NeuN) antibody staining; (**C**) 4′,6-diamidino-2-phenylindole (DAPI) nuclear staining; (**D**) Merged image of (**B**,**C**). All cells stained positive with the anti-NeuN antibody.

**Figure 4:**
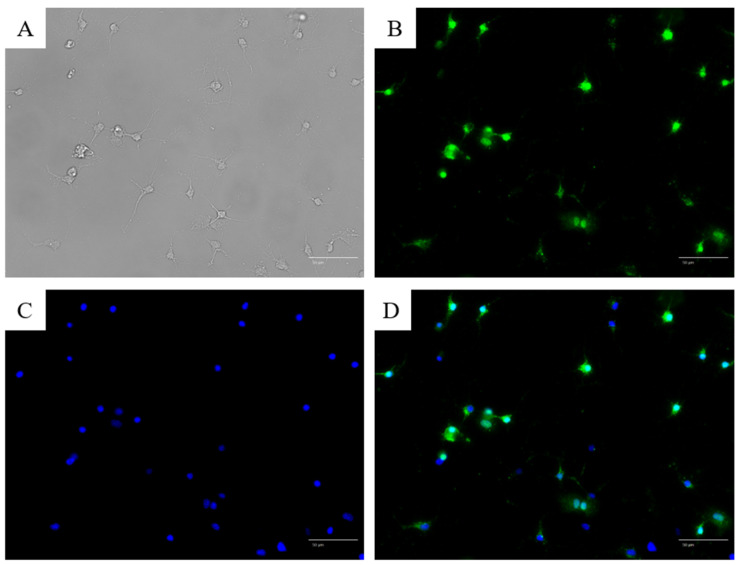
Microscopic images of trigeminal ganglion cells stained with anti-TRPV1 (monolabel). (**A**) Bright-field image showing neuron-like morphology with one thick axon and several dendritic branches. (**B**) TRPV1 immunofluorescence signal (green) showing cytoplasmic localization in a subset of neurons. (**C**) Nuclear counterstaining with DAPI (blue). (**D**) Merged image of (**B**,**C**).

NeuN is a marker of postmitotic neurons. NeuN antibody staining revealed green nuclei, indicating NeuN positivity and confirming that the cultured cells were neurons (Figure 3A–D). Further, anti-TRPV1 antibody staining revealed that a subset of cultured cells expressed the nociceptor-specific TRPV1 (Figure 4).

Although double immunostaining with NeuN and TRPV1 was not performed in this experiment, the neuronal identity of cultured cells was confirmed separately by NeuN staining (Figure 3), and the TRPV1 expression pattern was consistent with previous reports in trigeminal neurons.

### 3.3. Calcium Imaging of Primary Trigeminal Ganglion-Derived Cultured Cells (TRGCs)

**Figure 5:**
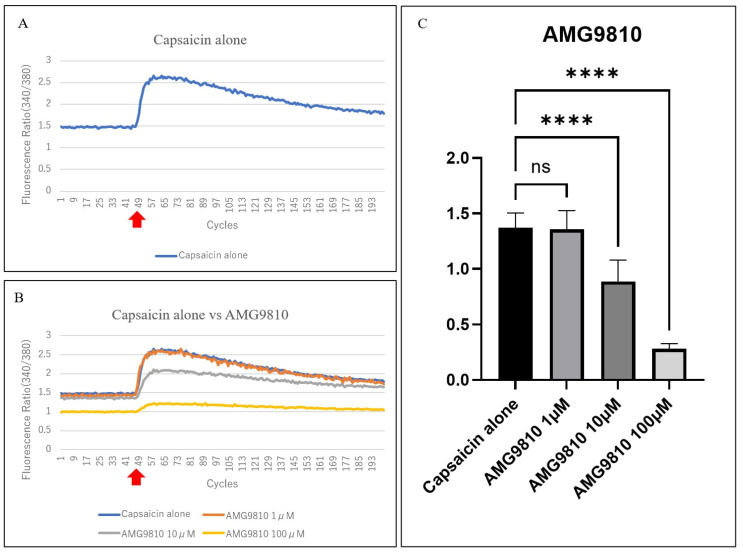
Comparison of the effects of capsaicin and the TRPV1 antagonist AMG9810 on TRGCs. The vertical and horizontal axes of the line graph show the 340 nm/380 nm fluorescence intensity ratio and the number of measurements (cycles), respectively. Cycles 1–47 were the baseline, and capsaicin was injected at cycle 48. Additionally, Δ340 nm/380 nm was defined as the maximum value of the 340 nm/380 nm fluorescence intensity ratio minus the mean baseline value. (**A**) Change in the fluorescence intensity ratio (340 nm/380 nm) over time before and after the addition of capsaicin. Capsaicin (10 μM) was added to stimulate cells approximately 1 min after the start of measurement (cycle 48, red arrow). The 340 nm/380 nm fluorescence intensity ratio increased when cellular excitation occurred. The mean 340 nm/380 nm ratio before capsaicin stimulation (red arrow) was used as the baseline, and the maximum 340 nm/380 nm ratio after capsaicin stimulation in cycle 48 was recorded. The difference between the maximum and baseline values was defined as Δ340 nm/380 nm; (**B**) Comparison of the change in the fluorescence intensity ratio (340 nm/380 nm) over time before and after the addition of capsaicin or AMG9810 (1 µM (*n* = 6), 10 µM (*n* = 16), and 100 µM (*n* = 14)) and capsaicin. Capsaicin alone (blue line (*n* = 17)) served as the no-addition group, and the other lines show the 340 nm/380 nm ratio before and after capsaicin stimulation for each AMG9810 concentration. The ratio increased rapidly after capsaicin stimulation (red arrow) in all groups. Compared with capsaicin alone, 1 µM AMG9810 showed little change; however, 10 µM and 100 µM of AMG9810 suppressed Δ340 nm/380 nm; (**C**) Δ340 nm/380 nm was significantly inhibited at 10 µM and 100 µM of AMG9810 compared with capsaicin alone. *p* > 0.999, *p* < 0.0001, and *p* < 0.0001 for 1 µM, 10 µM, and 100 µM of AMG9810, respectively; Dunnett’s multiple comparisons test; ns: not significant. Error bars indicate standard deviation (SD). ns, not significant; **** *p* < 0.0001.

We found that TRGCs could be excited after capsaicin stimulation (Figure 5A,B red arrow). Capsaicin induced a rapid increase in the 340/380 fluorescence intensity ratio, confirming TRPV1-mediated neuronal excitation (Figure 5A). The fluorescence intensity at 340 nm was also elevated because of the increase in Fura2-AM^®^ bound to calcium. In contrast, the fluorescence intensity of Fura2-AM^®^ alone decreased at 380 nm, causing an increase in the 340 nm/380 nm fluorescence intensity ratio. AMG9810 inhibited this response in a concentration-dependent manner, with significant suppression at 10 and 100 µM (both *p* < 0.0001), whereas 1 µM showed minimal effects (Figure 5A–C).

### 3.4. Evaluation of the Suppression of Capsaicin-Related Excitation in Primary TRGCs with Commercially Available Dry Eye Medications

**Figure 6:**
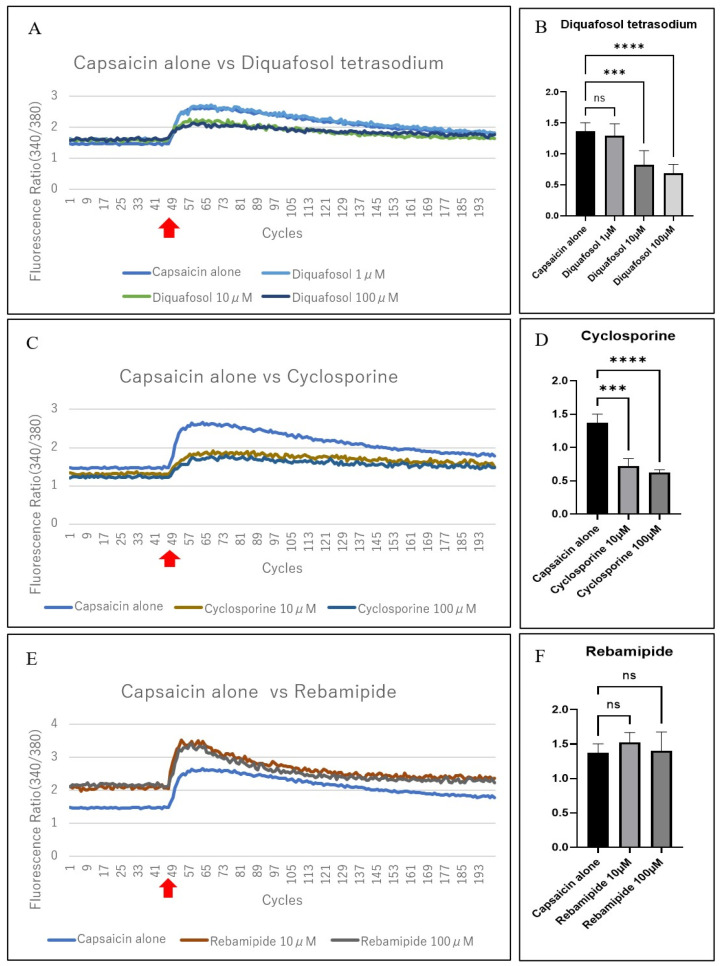
Comparison of the effects of capsaicin and commercially available eye drops on TRGCs. (**A**) Comparison of the change over time before and after the addition of capsaicin alone or diquafosol tetrasodium (1 µM (*n* = 8), 10 µM (*n* = 8), and 100 µM (*n* = 8)) plus capsaicin. Little difference was observed with 1 µM diquafosol tetrasodium compared with capsaicin alone; however, 10 and 100 µM diquafosol tetrasodium suppressed Δ340 nm/380 nm; (**B**) Δ340 nm/380 nm was substantially inhibited with 10 and 100 µM diquafosol tetrasodium compared with capsaicin alone. *p* = 0.67, *p* < 0.001, and *p* < 0.0001 for 1, 10, and 100 µM, respectively; Dunnett’s T3 multiple comparisons test; (**C**) Comparison of the change over time after the addition of capsaicin alone or cyclosporine (10 µM (*n* = 4) and 100 µM (*n* = 4)) plus capsaicin. Compared with capsaicin alone, 10 and 100 µM cyclosporine suppressed Δ340 nm/380 nm; (**D**) Δ340 nm/380 nm was substantially inhibited by 10 and 100 µM cyclosporine compared with capsaicin alone. *p* < 0.001 and *p* < 0.0001, respectively; Dunnett’s multiple comparisons test; (**E**) Comparison of the change over time before and after the addition of capsaicin alone or rebamipide (10 µM (*n* = 4) and 100 µM (*n* = 4)) plus capsaicin. Both concentrations of rebamipide showed little difference compared with capsaicin alone; (**F**) No substantial difference from capsaicin alone was observed with 10 or 100 µM rebamipide. *p* = 0.20 and *p* = 0.97, respectively; Dunnett’s multiple comparisons test. ns: not significant; Error bars indicate standard deviation (SD). Δ340/380 was calculated as the maximum fluorescence ratio minus the mean baseline value for each individual sample (**B**,**D**,**F**). ns, not significant; *** *p* < 0.001; **** *p* < 0.0001. Red arrows indicate capsaicin stimulation.

We investigated whether commercially available dry eye medications containing diquafosol tetrasodium, cyclosporine, or rebamipide, suppressed the capsaicin-induced excitation of TRGCs. Diquafosol tetrasodium and cyclosporine significantly reduced capsaicin-induced Δ340/380 at concentrations ≥10 µM (*p* < 0.001), while rebamipide showed no notable inhibitory effect at either tested concentration (Figure 6A–F). Diquafosol tetrasodium (10 and 100 µM) suppressed neural excitation following capsaicin stimulation (Figure 6A); however, no suppression was observed with 1 µM diquafosol tetrasodium (Figure 6B). Capsaicin-induced excitation was significantly suppressed by the addition of 10 or 100 µM cyclosporine (Figure 6C,D). In contrast, 10 or 100 µM of rebamipide did not influence capsaicin-induced excitation (Figure 6E), with no significant differences at either concentration (Figure 6F). A summary of these results is presented in Supplementary Table S1 online.

**Figure 7:**
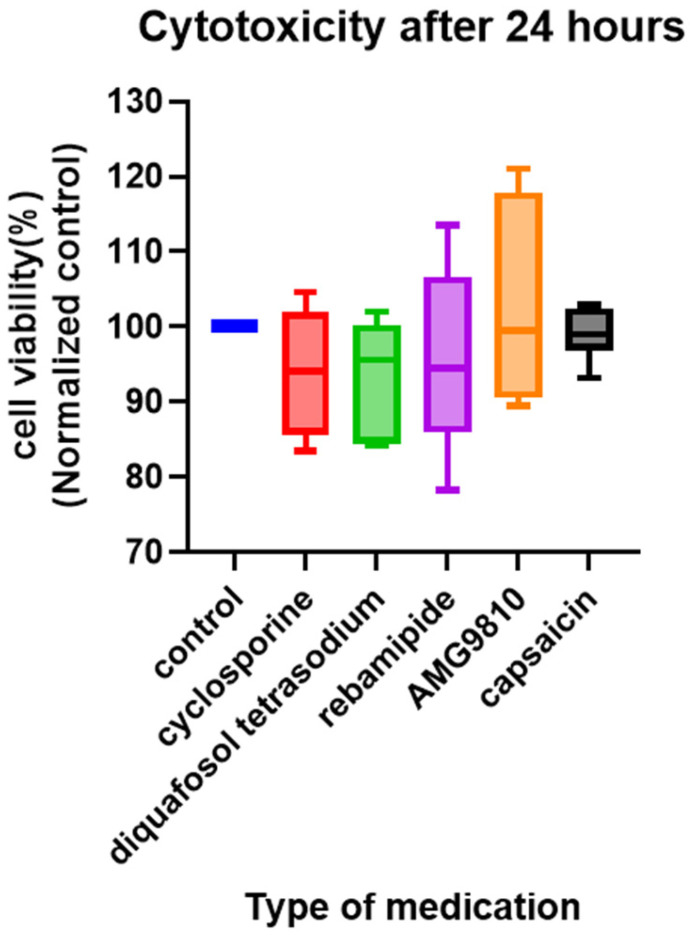
Viability of TRGCs exposed to various compounds. None of the compounds exhibited cytotoxicity against TRGCs. The control group consisted of untreated cells. All groups were cultured for 24 h. The sample size for each group was *n* = 6.

No cytotoxicity was observed with any medication under the tested conditions (Figure 7).

## 4. Discussion

In this study, we successfully used primary neuronal cell cultures from TRPV1-expressing corneal polymodal nociceptors obtained from postnatal day 7 mice to demonstrate increased intracellular calcium ions following capsaicin (a TRPV1 agonist) treatment. To our knowledge, this is the first study to evaluate mouse TRGC responses using a calcium ion probe after stimulation with clinically used medications to treat dry eyes. We found that diquafosol tetrasodium or cyclosporine, but not rebamipide, suppressed cellular excitation in TRGCs. Therefore, our findings suggest that the inhibition of cellular excitation by clinical agents may be associated with clinical pain suppression as patients with dry eyes demonstrate corneal pain sensitivity [33] and foreign body sensation and/or pain on the ocular surface.

Using bright-field microscopy, we observed that the TRGC cultures from postnatal day 7 mice showed a neuron-like morphology, including dendrites and axons. The neuronal identity of these cultures was further confirmed by NeuN immunostaining, as shown in Figure 3. To our knowledge, few studies have shown clear pictures of cultured mouse neurons using brightfield microscopy and positive staining for the neuronal marker NeuN. However, despite repeated attempts, we could not culture primary sensory neurons from TRGCs derived from postnatal day 1 mice or those older than postnatal day 12, suggesting technical difficulties in the culture of TRGCs derived from mice. Therefore, immortalized cells with gene transfer would contribute to further development of research using this experimental system.

Rebamipide and diquafosol tetrasodium have mucin-inducing and secretion-promoting effects [34]. However, our results demonstrated differential mechanisms of these products in relieving pain in patients with dry eye. Diquafosol tetrasodium and cyclosporine suppressed the capsaicin-induced excitation of TRGCs, while rebamipide did not. Nevertheless, the exact suppressive mechanisms of diquafosol tetrasodium and cyclosporine remain unclear.

Diquafosol tetrasodium is an agonist of the P2Y2 receptor that promotes corneal epithelial damage repair [35] and is expressed in various parts of the eye. P2Y2 agonists, including diquafosol tetrasodium, have also been reported to affect tumor necrosis factor receptor 1 (TNFR1) [36]. These results suggest that diquafosol tetrasodium binds to the P2Y2 receptor, causing TNFR1 release into the extracellular space, thus suppressing TNF-α signaling that may be involved in suppressing neural excitement through capsaicin stimulation.

Cyclosporine is a calcineurin inhibitor and an immunosuppressive agent, suppressing production of cytokines such as interleukin 2 [37]. Furthermore, cyclosporine may have an indirect suppressive effect on the production of cytokines involved in the cellular excitation of cultured mouse TRGCs. Further studies are required to identify the mechanisms underlying cyclosporine suppression of capsaicin-induced excitation in cultured TRGCs.

Dry eye disease is associated with peripheral sensitization of corneal nociceptors, partly mediated by TRPV1 activation through tear hyperosmolarity and inflammatory cytokines [38]. Sensitized TRPV1 triggers the release of neuropeptides such as substance P and calcitonin gene-related peptide (CGRP) [39], which may amplify ocular discomfort via neurogenic inflammation. Our finding that cyclosporine and diquafosol suppress TRPV1-dependent neuronal excitation suggests a plausible mechanism for their reported improvement in ocular pain in clinical settings. However, our in vitro experimental system did not incorporate corneal epithelial and immune cell interactions or tear components, which are key modulators of sensory function in vivo.

This study has some limitations. Although we investigated the effects of diquafosol tetrasodium and cyclosporine on neuronal excitation in mouse TRGCs, the underlying molecular mechanisms remain unresolved. In vivo studies involving specific receptors and dynamic changes in cytokines using knockout mice or a dry eye mouse model are required to validate our in vitro findings [40]. Clinical studies using eye drops and a Cochet–Bonnet esthesiometer [41] may also contribute to a better understanding of the mechanisms of pain development and reduction in pain in patients with dry eyes by commercially available eye drops. Furthermore, it remains unclear whether the suppression of cellular excitation was due to changes in calcium kinetics or the toxicity of the drugs added to the cultured cells. Furthermore, this study is limited by the absence of physiological microenvironmental factors, including epithelial–neuronal–immune cell crosstalk and tear film components. Future work involving quantification of neuropeptide release (e.g., CGRP, substance P) and correlating TRPV1 sensitivity with clinical corneal pain phenotypes using esthesiometry and in vivo confocal microscopy will help elucidate underlying mechanisms.

In conclusion, we used a primary cell culture model of TRGCs derived from postnatal day 7 mice that allowed the screening of medications that may affect nociceptive and neuropathic pain in the cornea. Our findings indicate that certain components of dry eye medications may act via inhibition of neuronal excitation. This model may serve as a powerful tool for future studies on corneal perception.

## Data Availability

All data generated or analyzed during this study are included in this published article and in its Supplementary Information Files.

## Supplementary Materials

The following supporting information can be downloaded at: https://www.mdpi.com/article/10.3390/jcm14228038/s1, Table S1: Summary of Results.

## Abbreviations

The following abbreviations are used in this manuscript:

PTGCs	Primary trigeminal ganglion cell cultures
TRGCs	Trigeminal ganglion-derived cells
TRPV1	transient receptor potential vanilloid 1
