# Supplementary material for: Evaluation of the Effects of Eye Drops for Dry Eyes on Neuronal Pain Receptors in a Primary Culture Model of Trigeminal Ganglion Cells
Source: J Clin Med. 2025 Nov 13;14(22):8038. doi: 10.3390/jcm14228038 (PMC12653758; doi:10.3390/jcm14228038)
Supplement: Supplementary file 1 [file jcm-14-08038-s001.zip › jcm-3967614-supplementary.pdf]

Supplementary Table S1

Summary of the Results

Drugs	Cell excitation after capsaicin stimulation
Diquafosol tetrasodium	inhibited
Cyclosporine	inhibited
Rebamipide	Uninhibited
AMG9810 (TRPV1 antagonist)	inhibited

TRPV1: transient receptor potential vanilloid 1
